# Correlation between Objective and Subjective Assessment of Nasal Patency

**Published:** 2016-09

**Authors:** Francesco Mozzanica, Roberto Gera, Chiara Bulgheroni, Federico Ambrogi, Antonio Schindler, Francesco Ottaviani

**Affiliations:** 1 *Department of Biomedical and Clinical Sciences, L. Sacco University Hospital, Milan, Italy.*; 2 *Division of Otorhinolaryngology, San Giuseppe Hospital, IRCCS Multimedica, University of Milan, Milan, Italy.*; 3 *Department of Clinical Sciences and Community Health, University of Milan, Milan, Italy.*

**Keywords:** Nasal Obstruction, Nose Diseases, Questionnaires, Rhinomanometry

## Abstract

**Introduction:**

This study was performed to evaluate the correlation between the objective and subjective sensation of nasal patency, assessed through a validated questionnaire, the Italian version of the NOSE scale, and the rhinomanometric results in a large cohort of patients complaining about nasal obstruction.

**Materials and Methods:**

Data was obtained from a total of 233 adult patients, (123 males, 110 females, with a mean age of 43.7 years) with a diagnosis of septal deviation and complaining about nasal obstruction. Anterior active rhinomanometry was used for objective assessment, while the I-NOSE scale and a visual analog scale (VAS) were used for subjective evaluation.

**Results:**

Positive correlations between I-NOSE scores and VAS and rhinomanometric results were found. The higher correlation was demonstrated between the HUNR (higher unilateral nasal resistance) parameter of rhinomanometry and the second item of the I-NOSE scale (Nasal blockage or obstruction). No significant correlation was found between the fourth item of the I-NOSE (Trouble sleeping) and the VAS score. The VAS score appeared mildly, but still significantly, correlated with the HUNR parameter of rhinomanometry.

**Conclusion:**

The correlation between the subjective sensation of nasal patency and the rhinomanometric data proved to be significant. No correlation between subjective sensation of trouble sleeping and rhinomanometric assessment was found. In counselling with patients complaining of nasal obstruction trouble in sleeping should not be considered as a symptom related to nasal obstruction.

## Introduction

Nasal obstruction sensation is a common symptom in otorhinolaryngology practice and is often reported as a feeling of insufficient airflow through nasal cavities (1). Nasal obstruction can have several aetiologies, such as turbinate hypertrophy, nasal polyps, deviation of the nasal septum, and others (2). Regardless of the aetiologies or treatments used, a thorough assessment of nasal obstruction remains a matter of debate because its objective assessment is controversial and there is no agreement on an accepted measurement tool (2-5). While the sensation of nasal obstruction is measured through validated questionnaires or visuo-analogue scales (VAS), its objective measurement may be accomplished through rhinomanometric techniques or radiological imaging (6). Although subjective and objective measurements are two different constructs, some correlations are expected as both constructs are related to nasal obstruction. 

Several authors compared the subjective sensation of nasal patency with objective and diverging results (2) (Table.1). 

**Table 1:** Previous studies analyzing the correlation between subjective sensation of nasal obstruction assessed through VAS or NOSE scale and nasal airway resistance measured through active anterior rhinomanometry. For each study the results of the correlation and the statistical analysis used are reported

**Study**	**Number**	**Statistical analysis**	**Symptom evaluation**	**Results**
Jones et al(7)	250 subjects	Spearman Rank correlation	VAS	r = -0.064
Sipila et al (9)	200 patients	k coefficient	VAS	k ranging from 0.417* to 0.700*
Simola et al (22)	101 patients	Pearson correlation test	VAS	r = 0.377*
Kim et al (8)	32 patients	Pearson correlation test	VAS	r = 0.25
Szucs et al (23)	50 patients	Spearman Rank correlation	VAS	not reported
Numminem et al (10)	69 patients	Pearson correlation test	VAS	r < 0.40*
Tompos et al (12)	86 patients	Spearman Rank correlation	VAS	r = -0.241*
Ng et al (11)	101 patients	Simple regression	VAS	[β] = 0.74; 95% CI: 0.27-1.21* [β] = 0.95; 95% CI: 0.26-1.64*
Menger et al (6)	34 patients	Pearson correlation test	NOSE	r = 0.26
Mozzanica et al(16)	60 patients	Spearman Rank correlation	NOSE VAS	r = 0.639* not assessed
Hsu et al (20)	50 patients	Pearson correlation test	NOSE VAS	r = -0.263 r = -0.165

* = presence of a statistical significant correlation

In particular Jones et al and Kim et al reported no correlation between total nasal resistance, assessed through rhinomano- metry, and symptoms of nasal obstruction. Sipila et al, Numminem et al, Ng et al, and Tompos et al reported mild correlations between subjective analysis of nasal patency and nasal airway resistance (7-12). Moreover, in a recent review André et al concluded that the correlation between objective assessment of nasal patency (evaluated through rhinomanometry and acoustic rhinometry) and subjective assessment was uncertain and consequently only limited arguments for the use of these objective measures in routine clinical practice existed (2). 

It is possible that these diverging results might be related to the tool used for subjective assessment of nasal patency. In previous studies, for the most part, validated questionnaires or VAS scales were not used and it is possible that these instruments were not sufficiently specific in order to assess the symptoms related with nasal obstruction (13). Recently, a new symptom specific questionnaire has been developed, the NOSE scale (3). This scale is a simple and fast questionnaire structurally composed of five obstruction-related items which evaluate the severity of complaints that the patient has been experiencing over the past month. While item 1 (Nasal congestion or stuffiness), 2 (Nasal blockage or obstruction), 3 (Trouble breathing through my nose) ask about symptoms directly related to nasal obstruction, item 4 (Trouble sleeping) and 5 (Unable to get enough air through my nose during exercise or exertion) investigate indirect consequences of nasal obstruction, such as trouble sleeping or breathing difficulties during exercise. All items are scored using a five points Likert scale where higher scores mean greater nasal obstruction. The NOSE scale has been adapted and validated in several languages, including Italian (14-16) and has been used in different outcome studies (17-21). However, to the best of our knowledge, only limited information regarding the correlation between NOSE scores and objective measurements of nasal patency analysed through anterior nasal rhinomanometry are available (6,16,20). In addition, the data reported so far is controversial. Hsu et al did not find significant correlations between NOSE scores and total nasal resistance assessed preoperatively or postoperatively in a group of 50 patients who underwent septoplasty for nasal septal deviation (20). Also Manger et al did not report significant correlation between the NOSE scores and the rhinomanometric results in 34 patients undergoing surgery on the external nasal valve (6). On the other hand, Mozzanica et al reported significant correlations between NOSE scores and rhinomanometric results in a group of 60 patients with nasal obstruction (16). It is possible that these diverging results might be related to the relatively small population recruited, which never exceed the number of 60 patients (16). In order to obtain a more robust evidence regarding the correlation between subjective and objective assessment of nasal patency, the correlation between rhinomanometric results and NOSE scores were analysed in a large group of patients complaining of nasal obstruction. In addition, the correlation of each item of the NOSE scale with the rhinomanometric results were analysed. This information might be useful in clinical practice since the knowledge of which symptoms correlate more with objective measurement could help clinicians in the evaluation of outcomes and in the pre- and post-treatment counselling in patients complaining about nasal obstruction. 

## Materials and Methods

Clinical data was obtained from a total of 233 consecutive patients (123 males and 110 females) consulting for nasal obstruction. The median age of the participants was 43.9 years (range 18-77). All data were collected prospectively and each subject who enrolled in the study gave his written informed consent. Only patients with normal cognitive function and preserved reading skills were included. The study was carried out according to the Declaration of Helsinki and it was previously approved by the Institutional Review Boards of the involved hospitals.

Exclusion criteria were: sinonasal malignancy, radiation therapy to the head or neck region, septoplasty performed with concurrent sinus surgery, rhinoplasty, or sleep apnea surgery; septoplasty performed as access to other sites; prior septoplasty, rhinoplasty, or turbinoplasty; history or clinical evidence of chronic sinusitis, septal perforation, cranio-facial syndrome, acute nasal trauma or fracture in the past 3 months, nasal valve collapse, adenoid hypertrophy, sarcoidosis, Wegener’s granulomatosis, uncontrolled asthma, pregnancy, and illiteracy (12,16). Inclusion criteria were: at least 18 years of age, presence of a septal deviation (diagnosed by CT scan or by rigid endoscopy) consistent with the reported symptoms, symptoms lasting for at least 3 months, and persistent symptoms after a 4-week trial of medical management, including either topical nasal steroids, topical or oral decongestants, or oral antihistamine-decongestant combina- tions (11,15).

Each of the enrolled patients was evaluated using anterior nasal rhinomanometry (Ryno-Zig, Menfis bioMedica, Bologna, Italy). Anterior nasal rhinomanometry is a dynamic test of nasal function that calculates the nasal Resistance (NAR) by measuring the trans-nasal pressure and airflow through nostrils during respiration. All measurements were performed by the same clinician under the same standard conditions, in compliance with the recommendations of the International Standardization Committee for Rhinomanometry (24). The decongestion of the nasal mucosa was not used. The sample pressures for unilateral measures was 150 Pa. Nasal resistance was measured in units of Pa/s/cm^-3^. Similar to the Tompos et al study (11), five dependent variables were considered during the evaluation of the anterior nasal rhinomanometry results: right nasal resistance (RNR), left nasal resistance (LNR), total nasal resistance (TNR), higher unilateral nasal resistance (HUNR), and lower unilateral nasal resistance (LUNR), each recorded at sample pressures of 150 Pa. In order to assess the subjective sensation of nasal obstruction, before the rhinomanometric analysis, each patient managed to autonomously complete the I-NOSE scale (15). Similarly to the study by Stewart et al (3), a VAS measuring the subjective sensation of nasal obstruction was provided. A 100-mm line with the extremes “nose feels extremely blocked” (100 mm) and “nose feels extremely clear” (0 mm) was used. The clinician who performed the anterior nasal rhinomanometries did not consider the I-NOSE and VAS results. Statistical tests were performed using SPSS 20.0 (SPSS, Inc., Chicago, IL). The sample size allowed the detection of a moderate correlation. In fact, a sample size of 233 produces a two-sided 95% confidence interval with a width equal to 0,234 when the sample correlation is 0,300 (95% CI: 0,178-0,413). Considering a correlation of 0.500 the two-sided 95% confidence interval would be 0,194. The correlation between I-NOSE scores and rhinomanometric results was assessed using Pearson test. The same test was also used to evaluate the correlations between I-NOSE, VAS scores, and age. The distribution of I-NOSE scores in male and female subjects with nasal obstruction was evaluated using the Student t-test.

## Results

All patients managed to autonomously complete the I-NOSE scale in less than 3 minutes. The mean total I-NOSE score in patients complaining of nasal obstruction was 60.5 ± 23.2 (range 15-90). The mean total I-NOSE score for males was 61.8 ± 24.4, while for females it was 58.9 ± 22.5. These differences were not found to be significant on Student t-test (P= 0.58). In addition, no significant differences were found in the scores obtained in the different items of the I-NOSE between males and females (P= 0.51; P= 0.24; P= 0.64; P= 0.33; P= 0.19, for the items 1, 2, 3, 4 and 5, respectively). The mean VAS score was 49.8 ± 20.1 mm (range 5-91). The mean VAS score in males was 50.5 ± 21.2, while in females it was 48.1±19.8. These differences were not found to be significant on Student t-test (P= 0.81). In the group of patients, age was not significantly correlated to VAS score nor to the overall I-NOSE score and to each of its items.

Rhinomanometric results and the correlations with I-NOSE and VAS scores in the group of 233 patients who underwent active anterior rhinomanometry are reported in Table 2. The parameters with higher correlations with I-NOSE total scores was HUNR 150 (r= 0.540). As far as the single items of I-NOSE are concerned, the item with highest correlation with HUNR 150 was the second ("Nasal blockage or obstruction" r = 0.543), while the items with lower correlation were the fourth and the fifth ones ("Trouble sleeping" r=0.393, “Unable to get enough air through my nose during exercise or exertion” r = 0.474). Also VAS scores appeared significantly correlated with HUNR 150 (r= 0.372).

**Table 2:** mean ± standard deviation of anterior nasal rhinomanometry results and their correlations with I-NOSE and VAS scores. The ranges are reported in brackets

	**Mean ± SD**	**I-NOSE **						**VAS**
		Q1	Q2	Q3	Q4	Q5	Total	
RNR 150	0.83 ± 0.91 (0.16-3.81)	0.198	0.164	0.207	0.196	0.276	0.183	0.185
LNR 150	1.03 ± 1.09 (0.23-4.21)	0.331	0.319	0.369**	0.322*	0.347	0.412**	0.196
HUNR 150	1.46 ± 1.39 (0.33-4.21)	0.507**	0.543**	0.520**	0.393*	0.474**	0.540**	0.372*
LUNR 150	0.60 ± 0.67 (0.16-3.50)	0.119	0.342	0.226	0.313	0.282	0.164	0.138
TNR 150	0.40 ± 0.46 (0.10-2.50)	0.203	0.208	0.254	0.308	0.276	0.235	0.143

RNR = right nasal resistance, LNR = left nasal resistance, HUNR = higher unilateral nasal resistance, LUNR = lower unilateral nasal resistance, TNR = total nasal resistance, Q1-Q5 = item 1-5 of the NOSE scale.

* = P < 0.05

** = P < 0.01

The correlation between VAS and I-NOSE scores is reported in Table 3. No significant correlation was found between the fourth item of I-NOSE (“Trouble sleeping”) and VAS score while higher correlation was found between the third item of I-NOSE (“Trouble breathing through my nose”) and VAS score. 

**Table 3:** Correlations between I-NOSE total score and single item score and VAS scores

**I-NOSE **	**Item**	**Pearson**
1	Nasal congestion or stuffiness	0.54**
2	Nasal blockage or obstruction	0.58**
3	Trouble breathing through my nose	0.65**
4	Trouble sleeping	0.41
5	Unable to get enough air through my nose during exercise or exertion	0.59**

** = P< 0.01

## Discussion

In this study the correlation between subjective sensation of nasal patency (analysed through a validated questionnaire, the I-NOSE scale, and a VAS) and objective measurement (analysed through rhinomanometric results) in a large group of patients with septal deviation complaining of nasal obstruction was analysed. To the best of our knowledge, only a relatively small number of studies investigated the correlation between rhinomanometry and subjective sensation of nasal patency (2). In the majority of them, the subjective analysis of nasal patency was based on patient self-assessment with visual analogue scales and only a limited number of studies used the NOSE scale (6,16,20). The reported results appeared controversial and André et al concluded that only limited arguments for the use of objective measures in routine clinical practice existed since their correlation with subjective assessment was uncertain (2). Some authors, in fact, found no correlation between subjective analysis of nasal patency and nasal airway resistance (7,8). On the other hand, Sipila et al, Numminem et al, and Ng et al found mild but significant correlation between total nasal resistance and nasal obstruction symptoms (9-11). Tompos et al found weak but significant correlation between HUNR 75 and HUNR 150 and symptoms of nasal obstruction (12). Also in the present study significant correlation between VAS scores and rhinomanometric results were found. In addition, higher correlation was found between I-NOSE scores and rhinomanometric results. In particular, the items with highest correlation with rhinomanometric results were the first 3 items (Nasal congestion or stuffiness, Nasal blockage or obstruction, Trouble breathing through my nose respectively), while lower correlation was found for the fourth (Trouble sleeping) and the fifth item (Unable to get enough air through my nose during exercise or exertion). These findings are not surprising as both trouble in sleeping and feeling of nasal obstruction during exercise are not directly related to rhinomanometric measurements as item 1, 2 and 3 are. Different findings might be expected in patients complaining about nasal obstruction with an aetiology that differs from septal deviation. We might speculate that in patients, who complain of nasal obstruction and have a diagnosis of allergic rhinitis, a stronger correlation between item 4 and rhinomanometric measurement could be found. It is possible to speculate that the strong correlations found in this study between rhinomanometric results and the I-NOSE scores are related to the the sample size and the questionnaire itself. This I-NOSE scale is a patient-centred tool, specifically developed in order to assess the symptoms a person could complain of as a consequence of his disease. The rhinomanometric analysis on the other hand measures airflow nasal resistances and it is reasonable that high nasal resistances determine - as one of the most relevant symptom-a sensation of obstruction or of nasal blockage (16). As far as the correlation between I-NOSE and the VAS are concerned, the results here reported appear similar to those previously reported (3).

## Conclusion

These data further support the clinical applicability of the I-NOSE scale (rather than a VAS) and of the rhinomanometry in the subjective and objective evaluation of patients complaining of nasal obstruction. The significant correlations between subjective and objective measurements of nasal patency suggest that the simultaneous application of both these types of measurements may provide additional information in the assessment of patients complaining of nasal obstruction as well as in the evaluation of surgical and medical results on respiratory comfort. In addition, it appears that trouble in sleeping and difficulty to get air through the nose during exercise are not strongly correlated with objective assessment of nasal obstruction in patients with septal deviation. This information could be helpful in the pre- and post-operative counselling in patients complaining about nasal obstruction. 
